# cMetS Based on Z-Scores as an Accurate and Efficient Scoring System to Determine Metabolic Syndrome in Spanish Adolescents

**DOI:** 10.3390/jpm13010010

**Published:** 2022-12-21

**Authors:** Ángel Fernández-Aparicio, Javier S. Perona, Jacqueline Schmidt-RioValle, Miguel A. Montero-Alonso, Carmen Flores Navarro-Pérez, Emilio González-Jiménez

**Affiliations:** 1 Department of Nursing, Faculty of Health Sciences, Melilla Campus, University of Granada, 52005 Melilla, Spain; 2 Instituto de Investigación Biosanitaria (ibs.GRANADA), 18014 Granada, Spain; 3 Department of Food and Health, Instituto de la Grasa-CSIC, Campus of the University Pablo de Olavide, 41013 Seville, Spain; 4 Department of Nursing, Faculty of Health Sciences, University of Granada, 18016 Granada, Spain; 5 Department of Statistics and O.I., Faculty of Medicine, University of Granada, 18016 Granada, Spain; 6 Department of Nursing, Faculty of Nursing, Physiotherapy and Podiatry, University of Seville, 41009 Seville, Spain

**Keywords:** metabolic syndrome, continuous metabolic syndrome, adolescents, determination, criteria

## Abstract

The definition of metabolic syndrome (MetS) based on dichotomous cut-off points is efficient in the adult population. However, to date, there is no international consensus on how to define MetS in the pediatric population. For that reason, a continuous MetS score (cMetS) has been proposed for the pediatric population. However, despite multiple attempts, cMetS has not been fully validated as there is no agreement about the most accurate score to calculate it. The purpose of the present study was to compare the validity of different scores (three siMS scores, z-score, principal components analysis (PCA), the sum of PCA, and confirmatory factor analysis) to calculate cMetS and determine MetS in Spanish adolescents. There were 981 subjects, ranging 11–16 years old, recruited for this cross-sectional study. Seven different approaches to pediatric cMetS scores were calculated. All cMetS scores calculated strongly correlated with each other, especially siMS scores. The area under the curve obtained from receiving operating characteristic curves was particularly elevated for z-scores 0.81 (95% CI: 0.784–0.838), showing a specificity of 64.4%. Our study shows that cMetS based on z-scores is accurate and efficient to be used for research instead of the dichotomized definition of MetS in adolescents; and cMetS based on siMS scores is useful for clinical practice.

## 1. Introduction

Metabolic syndrome (MetS) is recognized as a common and multifactorial disorder that is strongly associated with an increased risk of developing atherosclerotic cardiovascular disease and type 2 diabetes [1]. In adults, MetS are frequently defined as a cluster of cardiometabolic abnormalities that includes central obesity, hypertension, insulin resistance, and atherogenic dyslipidemia [2].

Multiple observational studies have shown that the MetS starts with central obesity. Because the prevalence of obesity in the adolescent population has doubly increased worldwide in recent decades, the rates of the MetS in adolescents have markedly boosted [3]. However, there is no international consensus on how to define MetS in adolescents so far [4], mainly due to the lack of universal reference values for each of the MetS components in the adolescent population [5]. On the other hand, the definition of MetS based on dichotomous cut-off points has proven to be efficient in the adult population, but due to a lower prevalence (<10%) and fewer large-scale studies in adolescents [6], it may not be applicable for the diagnosis of MetS in the adolescent population. Therefore, the American Diabetes Association and the European Association for the Study of Diabetes have suggested using a continuous score for adolescents instead of a dichotomous score [7]. In order to conduct a continuous score for MetS, different studies have used approaches of Z-score, factorial analysis, principal components analysis (PCA), and confirmatory factor analysis (CFA) [8,9], being the continuous metabolic syndrome (cMetS) scores provided by these methods sample specifically [10]. Eisenmann et al. [11] were the first to study the validity of cMetS in the American adolescent population, demonstrating a strong relationship between cMetS values and the number of MetS components. Subsequently, other have employed cMetS scores in children and/or adolescents in Brazil [12], Korea [13], Iran [14], Finland [15], India [16], and Kuwait [17].

In spite of existing different approaches to calculating cMetS scores, their validity has not been examined in the Spanish adolescent population. To our knowledge, the only attempt in this population was carried out by Formisano et al. [18], who used cMetS to determine the efficacy of neck circumference for discriminating MetS in a collaborative study that included Spanish 3–10-year-old children. Therefore, the aim of this study was to compare the validity of seven approaches to calculate pediatric cMetS scores, including three siMS scores, Z-scores, PCA, the sum of PCA, and CFA for determining MetS in Spanish adolescents.

## 2. Materials and Methods

### 2.1. Participants and Study Design

**Figure 1:**
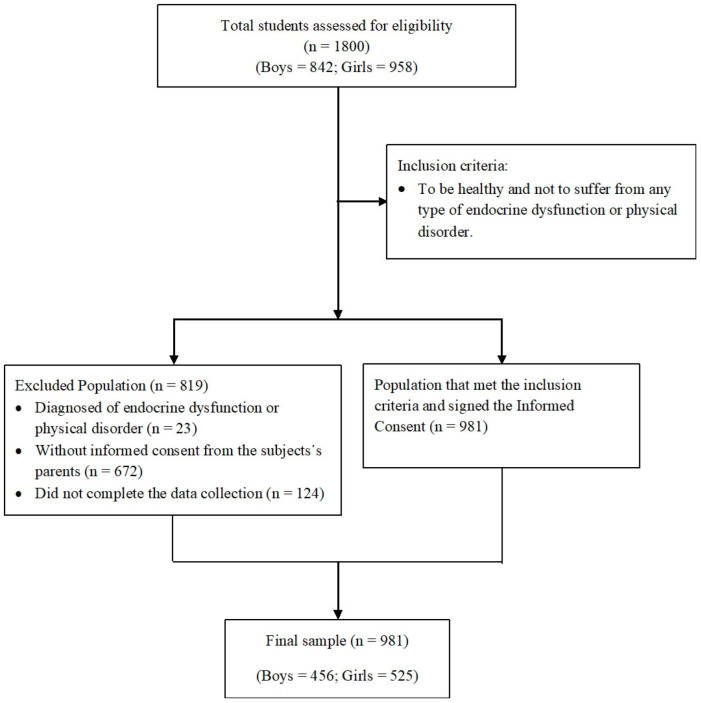
Flow diagram of the recruitment progress.

The present is a cross-sectional study that was performed on 981 adolescents (456 boys and 525 girls), 13.2 (1.1) years of age (ranging from 11–16 years old). All participants were of Spanish origin and had a similar socioeconomic status. For the recruitment process, purposive sampling was performed, in which principals of a total of 23 high schools in the provinces of Granada and Almeria were sent a letter of invitation, and only 18 schools agreed to participate in the study. In each school, two classes per grade were randomly selected to participate in the study. In order to participate in the study, the individuals needed to be healthy and were excluded if they presented any type of endocrine dysfunction or physical disorder. At the beginning of the study, informed consent was obtained from the subjects’ parents or guardians. The flow diagram (Figure 1) summarizes the recruitment process. The study had been previously approved by the Ethics Committee of the University of Granada and was authorized by either the Board of Education of the Andalusian Government or the school principals. This research was performed in strict compliance with the International Code of Medical Ethics (World Medical Association) and the Declaration of Helsinki.

### 2.2. Physical Measurements

Body mass index was calculated from height and weight, which were measured without shoes and with light clothing. Waist circumference (WC) was measured midway between the lower border of the rib margin and the iliac crest at the end of normal expiration. Systolic (SBP) and diastolic (DBP) blood pressures were measured twice using a standardized sphygmomanometer on the right arm after a 15-min rest in a sitting position; the first and fifth Korotkoff sounds were recorded as SBP and DBP, respectively. The mean of the two measurements was considered as the participant’s blood pressure. Mean arterial pressure (MAP) was calculated using the following formula: MAP = [(SBP − DBP)/3] + DBP [19].

### 2.3. Blood Sampling

A blood sample was drawn from participants after overnight fasting and analyzed on the same day. Total cholesterol (TC), high-density lipoprotein-cholesterol (HDL-C), triglycerides (TG), and fasting blood glucose (FBG) were measured enzymatically by means of a Hitachi auto-analyzer (Tokyo, Japan) [20].

### 2.4. Metabolic Syndrome Criteria According to the International Diabetes Federation (IDF)

MetS were diagnosed in adolescents, according to the International Diabetes Federation (IDF), when they fulfilled the following criteria: waist circumference ≥ 94 cm in boys and ≥80 cm in girls, as well as two of the following risk factors: FBG between 100–125 mg/dL, serum TG ≥ 150 mg/dL, HDL-C < 40 mg/dL in boys and <50 mg/dL in girls, and SBP/DBP ≥ 130/85 mmHg [21]. Therefore, subjects who met the above-mentioned criteria were classified as MetS and those not meeting the criteria as Non-MetS.

### 2.5. Calculation of cMetS Score

The cMetS score was calculated after standardizing the residuals (z-scores) for FBG, MAP, WC, TG, and HDL-C by regressing them on age and sex to account for age- and gender-related differences [22]. In order to calculate the z-scores, our cohort was used as our own reference population. MAP was used instead of SBP and DBP to avoid loading two BP variables in the calculation. HDL-C was multiplied by −1 because the standardized values are inversely related to the MetS risk. The cMetS score was calculated as the sum of the (z-scores) for the individual variables [23]. A higher cMetS score indicates a less favorable metabolic profile [23]. The results of the PCA and the factor analysis carried out are shown in supplementary file S1. The first PCA score was obtained from the first component of PCA based on the five variables, which are components of MetS (i.e., WC, HDL-C, TG, MAP, and FBG). The sum of PCA was calculated using the sum of components derived from PCA with eigenvalues greater than 1. The CFA score was obtained from performing CFA on the same variables. The siMS scores were computed as follows:

siMS score 1 = 2 × Waist/Height + FBG (mmol/L)/5.6 + TG (mmol/L)/1.7 + SBP/130 + HDL-C (mmol/L)/1.02

siMS score 2 = 2 × Waist/Height + FBG (mmol/L)/5.6 + TG (mmol/L)/1.7 + SBP/130 + HDL-C (mmol/L)/1.02 or 1.28 (male/female)

siMS score 3 = 2 × Waist/Height + FBG (mmol/L)/5.6 + TG (mmol/L)/1.2 + SBP/130 + HDL-C (mmol/L)/1.15 or 1.02 (15–18 years, boys/others)

All siMS scores are based on the IDF MetS criteria.

### 2.6. Statistical Analysis

Continuous variables were expressed as mean (standard deviation, SD), while categorical variables were expressed as numbers (percentage). For comparisons between boys and girls, the normality of the variables was assessed using the Shapiro-Wilk test, and Student’s *t*-test or Mann-Whitney test was used accordingly. In order to determine the association of the different cMetS between them, Pearson’s correlation coefficient was used. To compare the mean number of adverse risks, one-way ANOVA was used, and ROC curves [24] were employed to assess the overall performance of the different cMetS scores to discriminate between MetS or non-MetS adolescents, using the method of DeLong et al. [25] SPSS v25.0 (IBM, Armonk, NY, USA) [26] and R [27] statistical software were used for statistical analyses. The *p*-values of lower than 0.05 were considered statistically significant.

## 3. Results

981 adolescents aged between 11 and 16 years participated in this cross-sectional study, 53.51% of which were boys.

The mean age was 13.26 (1.15) and 13.18 (1.15) years for girls and boys, respectively. Table 1 shows the mean (SD) for weight, height, WC, BMI, FBG, TG, HDL, SBP, DBP, and MAP by gender. Boys showed significantly greater mean values for weight, height, WC, SBP, and MAP compared with girls (*p* < 0.05).

Table 2 shows the number of participants and cMetS means obtained from the different approaches by the number of adverse risk factors. There were 463 participants that had at least one adverse risk factor. Means increased significantly with an increasing number of components (*p* < 0.001).

Correlation coefficients between each cMetS score approach with each other are denoted in Table 3. cMetS scores showed strong and significant correlations with each other, especially among siMS scores. The lowest correlation was found for the Sum of PCA. The siMS scores showed the highest correlation with the z-score, followed by the CFA, and finally, with the first PCA score.

**Figure 2:**
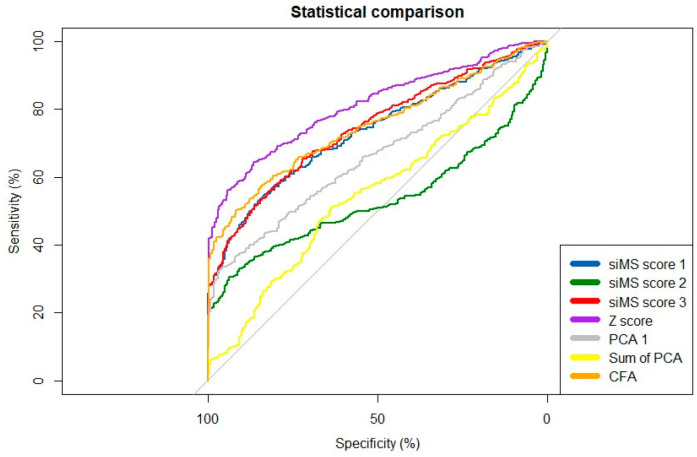
ROC curves for the continuous metabolic syndrome scores as discriminators of metabolic syndrome.

The results of ROC curve analyses, including the AUC values and their 95% confidence intervals (CIs) for cMetS scores, are presented in Table 4. AUC values for cMetS scores were substantially elevated for z-score and CFA. Nevertheless, the highest AUC for discriminating MetS was 0.81 (95% CI: 0.784–0.838) for the cMetS z-score, also showing a specificity of 64.4%. The differences in AUC values of cMetS scores were significant according to the De-Long method (*p* < 0.001). ROC curves are also depicted in Figure 2.

## 4. Discussion

To the best of our knowledge, the present is the first study to compare the validity of different cMetS scores using MetS risks in Spanish adolescents. These findings are relevant considering the existence of studies suggesting that cMetS scores are more reliable than the dichotomous definition of MetS for discrimination in adolescents [14,28,29]. Other studies also suggest that a continuous scale is statistically more sensitive and less error-prone compared to the dichotomous approach [30,31]. In this study, we found that the seven cMetS score approaches studied had different levels of accuracy for estimating the risk of MetS. Our results show that means of cMetS scores increased significantly with an increasing number of adverse risk factors, which agrees with Pandit et al. [16] in their study of 236 Indian adolescents. These findings indicate a graded relationship between cMetS and adverse risk factors of MetS [32].

On the other hand, a strong correlation of siMS scores with other continuous scores was observed, being the highest correlation with the z-score, then the CFA, and finally with the first PCA score. These findings are partially consistent with the results obtained by Vukovic et al. [33] in Serbian adolescents, who found that siMS calculated using formulas 1 and 2 had a close correlation with z-scores and the weighted sum of factors of PCA. In addition to these findings, one point to consider is that siMS scores (formulas 1–3) are simple to calculate and could be used for the follow-up of individual patients, and thus potentially useful in the clinical setting, unlike other cMetS scores such as z-scores, PCA and CFA analysis methods [34], which are only applicable to groups of patients, and therefore only useful for calculating cMets, as they are sample-specific methods.

The results of comparisons across AUCs obtained from seven cMetS scores indicated that cMetS based on z-scores had the highest predictive power (AUC = 0.811) for MetS in the adolescent population studied; it also had the highest percentage of specificity, which is consistent with the results obtained by Shi et al. [17]. Likewise, Okosun et al. [35], in their study of American adolescents, found that cMetS based on z-scores had a high predictive power for MetS (AUC > 0.885), being slightly higher than that obtained in our study. On the other hand, siMS score 3 showed predictive ability for MetS in the population studied (AUC = 0.745), which agrees with Lee et al. [36], who concluded that siMS score 3 has reliable predictive power for the identification of adolescents with MetS. However, it must be considered that the performance of AUCs analyses requires dichotomizing the cMetS values, implying the loss of information.

Nevertheless, according to Khoshhali et al. [14], referring to z-score, PCA and CFA, since they are sample-specific methods, it is not possible to compare within studies unless the demographic characteristics, data distribution, measures of central tendency, and variability are similar. In consequence, there is a need to develop more studies with these features. On the other hand, according to Soldatovic et al. [34], approaches to the siMS score can be compared between different studies and populations. While the other scores are calculated from regression of principal components analysis of a whole sample, siMS is calculated from a single equation that can be applied to individual subjects. This is especially important if we consider the absence of reference standards for these indicators among the Spanish adolescent population that allow comparability of results.

This study has some limitations and strengths that should be mentioned. The main limitation is the cross-sectional nature of the data, together with the fact that all participants are from the same geographic area and have the same socio-economic characteristics. Also, the sample size for our study was not calculated. However, our study is strengthened by the large sample size of adolescents who participated. Another strength of the present study is the high correlation demonstrated between the cMetS score approaches studied, which provides a key scientific knowledge base for future longitudinal studies.

## 5. Conclusions

Our study shows that cMetS based on z-scores is an accurate and efficient scoring system that can be used to determine MetS in adolescents, but only for research because it is a sample-specific method. However, data in our study have shown a strong correlation between z-scores and siMS scores. The latter could be useful in clinical practice since they are single formulas that can be applied to individual subjects. Moreover, further investigations are required to compare the validity of the ultimate net values of cMetS with the dichotomized definition of MetS in adolescent populations but also in other age groups.

## Figures and Tables

**Table 1:** Characteristics of participants by age and gender.

Variables	Mean (SD)			*p*-Value ^a^
	Total	Girls (*n* = 525)	Boys (*n* = 456)	
Age, years	13.2 (1.2)	13.3 (1.2)	13.2 (1.2)	0.282
Weight, kg	54.9 (12.7)	53.1 (11.0)	57.1 (14.1)	**<0.001**
Height, cm	160.1 (8.9)	158.2 (6.9)	162.4 (10.4)	**<0.001**
WC, cm	72.4 (10.8)	71.3 (9.6)	73.7 (11.8)	**<0.001**
BMI, kg/m^2^	21.3 (3.8)	21.1 (3.6)	21.5 (4.0)	0.140
FBG, mmol/L	4.8 (1.7)	4.7 (1.6)	4.8 (1.7)	0.613
TG, mmol/L	1.4 (0.6)	1.4 (0.5)	1.5 (0.7)	0.221
HDL, mmol/L	1.0 (0.1)	1.0 (0.1)	1.0 (0.1)	0.707
SBP, mmHG	118.2 (15.5)	116.9 (15.1)	119.6 (15.7)	**0.006**
DBP, mmHG	64.2 (9.0)	63.9 (8.8)	64.5 (9.3)	0.291
MAP, mmHG	82.2 (10.3)	81.6 (9.9)	82.9 (10.7)	**0.046**

SD, standard deviation; WC, waist circumference; BMI, body mass index; FBG, fasting blood glucose; TG, triglycerides; HDL, high-density lipoprotein; SBP, systolic blood pressure; DBP, diastolic blood pressure; MAP, mean arterial pressure. ^a^ *p* values were obtained using the Student’s *t*-test for independent samples.

**Table 2:** Comparison of cMetS scores across the number of adverse risk factors.

Variables	Number of MetS Components				*p*-Value ^a^
	Mean (SD)				
	0 (*n* = 239)	1 (*n* = 463)	2 (*n* = 165)	≥3 (*n* = 114)	
SiMS score 1	4.27 (0.15)	4.33 (0.19)	4.70 (0.32)	5.56 (1.14)	**<0.001**
SiMS score 2	4.27 (0.15)	4.18 (0.25)	4.57 (0.37)	5.42 (1.20)	**<0.001**
SiMS score 3	4.27 (0.16)	4.33 (0.19)	4.69 (0.32)	5.55 (1.15)	**<0.001**
Z-scores	−2.06 (1.17)	−0.98 (1.57)	1.87 (2.04)	5.60 (4.42)	**<0.001**
First PCA	−0.44 (0.64)	−0.30 (0.79)	0.71 (0.89)	1.13 (1.12)	**<0.001**
Sum of PCA	−0.09 (0.82)	0.03 (0.87)	0.13 (1.41)	−0.11 (1.10)	**0.077**
CFA	−0.84 (0.65)	−0.50 (0.84)	0.93 (0.91)	2.44 (1.60)	**<0.001**

cMetS, continuous metabolic syndrome; SD, Standard deviation; PCA, principal component analysis; CFA, confirmatory factor analysis. ^a^ *p* values were obtained by means of one-way ANOVA.

**Table 3:** Pearson’s correlation coefficients between seven cMetS scores approach each other.

Variables	siMS Score 1	siMS Score 2	siMS Score 3	Z Score	First PCA	Sum of PCA	CFA
siMS score 1	1						
siMS score 2	0.98 **	1					
siMS score 3	0.99 **	0.98 **	1				
Z-scores	0.91 **	0.89 **	0.90 **	1			
First PCA	0.34 **	0.35 **	0.34 **	0.55 **	1		
Sum of PCA	−0.059	−0.066 *	−0.060	−0.020	0.000	1	
CFA	0.85 **	0.84 **	0.84 **	0.97 **	0.71 **	−0.018	1

cMetS, continuous metabolic syndrome; SD, Standard deviation; PCA, principal component analysis; CFA, confirmatory factor analysis. * *p*-value < 0.05; ** *p*-value < 0.01.

**Table 4:** ROC curve analysis for cMetS scores as determinants of MetS.

CMetS	AUC	95% CI	Optimal Cutoffs	Sensitivity %	Specificity %
siMS score 1	0.733	0.702–0.765	4.414	85.4	53.0
siMS score 2	0.534	0.499–0.569	4.499	93.7	30.5
siMS score 3	0.745	0.713–0.776	4.381	80.3	57.7
Z-scores	**0.811**	0.784–0.838	−0.891	86.6	64.4
First PCA	0.661	0.626–0.696	0.546	96.2	33.0
Sum of PCA	0.547	0.507–0.587	0.044	63.6	51.1
CFA	**0.750**	0.719–0.780	−0.028	91.6	50.3

cMetS, continuous metabolic syndrome; SD, Standard deviation; PCA, principal component analysis; CFA, confirmatory factor analysis; AUC, area under the receiver operating characteristic curves.

## Data Availability

The data presented in this study are available on request from the corresponding author. The data are not publicly available due to maintaining the privacy of participants.

## Supplementary Materials

The following supporting information can be downloaded at: https://www.mdpi.com/article/10.3390/jpm13010010/s1, Supplementary file S1: results of the PCA and the factor analysis.
