# Supplementary material for: cMetS Based on Z-Scores as an Accurate and Efficient Scoring System to Determine Metabolic Syndrome in Spanish Adolescents
Source: J Pers Med. 2022 Dec 21;13(1):10. doi: 10.3390/jpm13010010 (PMC9865991; doi:10.3390/jpm13010010)
Supplement: Supplementary file 1 [file jpm-13-00010-s001.zip › jpm-2016604-supplementary.pdf]

RESULTS OF THE ANALYSES

1. The first principal component analyses (PCA) score obtained from first component of PCA based on WC, MAP, HDL-C, TG and FBG

2. The sum of the PCA was calculated using the sum of components derived from PCA with eigenvalues greater than 1 (weighted for variance explained)

PCA

The most relevant results that justify the analyzes carried out are presented below. The following results are obtained when performing the Factor Analysis by the Principal Components method with SPSS. We obtained the correlation matrix with the significance of each component. Later, Barlett's test of Sphericity and Kaiser-Meyer-Olkin (KMO) measure of sampling adequacy were carried out.

Correlation Matrix[a]

		Fasting blood glycemia (mmol/L)	Triglycerides (mmol/L)	HDL-C mmol/L	Mean Arterial Pressure	Waist circumference
**Correlation**	**Fasting blood glycemia (mmol/L)**	1.000	0.718	- 0.737	- 0.006	0.035
	**Triglycerides (mmol/L)**	0.718	1.000	- 0.535	- 0.017	0.018
	**HDL-C (mmol/L)**	- 0.737	- 0.535	1.000	0.001	- 0.042
	**Mean Arterial Pressure**	- 0.006	- 0.017	0.001	1.000	0.542
	**Waist circumference**	0.035	0.018	- 0.042	0.542	1.000
**Sig. (unilateral)**	**Fasting blood glycemia (mmol/L)**		0.000	0.000	0.420	0.134
	**Triglycerides (mmol/L)**	0.000		0.000	0.303	0.284
	**HDL-C (mmol/L)**	0.000	0.000		0.487	0.095
	**Mean Arterial Pressure**	0.420	0.303	0.487		0.000
	**Waist circumference**	0.134	0.284	0.095	0.000	

a. Determinant = 0.155

KMO and Bartlett test

**Kaiser-Meyer-Olkin Measure of Sampling Adequacy**		0.627
**Bartlett's Test of Sphericity**	**Approx. Chi-square**	1819.320
	**df**	10
	**Sig.**	0.000

Barlett's and KMO test that the analysis performed is correct. Next, the correlation matrix was auto-decomposed into its eigenvalues and eigenvectors that reach the factor solution.

Total Variance Explained

Component	Initial Eigenvalues			Extraction Sums of Squared Loadings			Rotation Sums of Squared Loadings		
	Total	% of variance	Cumulative %	Total	% of variance	Cumulative %	Total	% of variance	Cumulative %
1	2.333	46.667	46.667	2.333	46.667	46.667	2.332	46.644	46.644
2	1.541	30.822	77.489	1.541	30.822	77.489	1.542	30.845	77.489
3	0.467	9.335	86.824						
4	0.455	9.107	95.931						
5	0.203	4.069	100.000						

Extraction Method: Principal Component analysis.
